# Oxidation Driven Damage on SiC/BN/SiC Ceramic Matrix Composite Aero-Engine Structures: An Iterative Computational Framework

**DOI:** 10.3390/ma17123034

**Published:** 2024-06-20

**Authors:** Giacomo Canale, Roberto Citarella

**Affiliations:** 1 College of Science and Engineering, Nuclear Engineering, University of Derby, Markeaton Street Campus, Derby DE22 3AW, UK; 2 Department of Industrial Engineering, Mechanical Engineering, University of Salerno, 84084 Fisciano, Italy; rcitarella@unisa.it

**Keywords:** CMCs, oxygen embrittlement, high-pressure turbine

## Abstract

Ceramic matrix composites (CMCs) could be a game changer in the aero-engine industry. Their density is circa one-third of their metallic counterpart. CMCs, furthermore, offer increased strength and greater capability at very high temperatures. This would allow for a reduction in cooling and an increased engine performance. Some challenges, besides the complexity of the manufacturing process, however, remain for the structural integrity of this technology. CMCs are inherently brittle; furthermore, they tend to oxidise when attacked by water or oxygen, and their constituents become brittle and more prone to failure. There are two main points of novelty proposed by this work. The first one is to model and reproduce recent oxidation experimental data with a simple Fick’s law implemented in Abaqus. The parameters of this modelling are a powerful tool for the design of such material systems. The second aspect consists in the development of a new computational framework for iteratively calculating oxygen diffusion and stiffness degradation of the material. Oxidation and stiffness degradation are in fact coupled phenomena. The crack (or microcracking) opening, the function of applied stress, accelerates oxygen diffusion whilst the oxidation diffusion itself contributes to embrittlement and then damage introduction in the material system.

## 1. Introduction

Operational requirements of the structural component on an aero-engine turbine sub-system are severe. Rotating components, such as turbine blades, are subjected to high stresses induced by the centrifugal forces and the gas pressures [1,2,3]. Both static components, such as vanes and seal segments, and rotating components, such as blades, are subjected to a severe thermal field and a corrosive environment. Temperature gradients can be as severe as hundreds of degrees Celsius per mm [4], whilst an oxygen- and water-rich environment can cause chemical reactions and challenge the structural performance of the materials. CMCs, with their reduced weight compared to Nichel Superalloys and their higher resistance to extreme temperatures (up to 1400 degrees Celsius), may help reduce the cooling system and improve weight and performance, reducing the specific fuel consumption [5]. Let the attention of this work be focussed on a specific component: a high-pressure turbine seal segment made of CMCs. This component, mounted to the high-pressure turbine casing, is fundamental to control the tip clearance of the turbine blade (the gap between the tip of the blade and the casing) and therefore the performance of the aero-engine [3]. The seal segment is loaded with pressures and temperature gradients, which are a function of the flight time. The component is coated, as a thermal barrier is needed as well as an environmental barrier, even if challenges such as TGO and CMAS still remain [6]. Not all the surface of a seal segment (or any other high-pressure turbine component) may be coated. The CMC can therefore be subjected to oxygen and water ingress. Oxygen attacks carbon in a C/SiC CMC and attacks carbon and boron nitrate in a SiC/BN/SiC [6] CMC. The chemical reaction is present both on the surface and in the interior of the material system. The chemical reaction in the interior part of the composite is facilitated by the presence of cracks, exaggerated by the presence of stress, voids, and porosity. Oxidation diffusion models have been already proposed by Medford for carbon–carbon space applications [7]. Halbig proposed an oxidation model in order to predict fibre recession in a C/SiC material system [8]. Chen et al. [9] have proposed a coupled model where the oxidation diffusion is coupled with the stress field. A complete thermo-chemical–mechanical fully coupled model has been proposed by Zhao et al. [10] for assessing the failure of CMCs. A mathematical framework for predicting the oxidation behaviour of carbon silicon carbide CMCs has also been developed by Sullivan [11]. The reaction rate after the diffusion of oxygen in a porous mean is calculated with an Arrhenius-type equation, the function of the local partial pressures of the gas components and local temperatures.

The first part of the work proposed in this article is to calibrate the experimental absorption data with a simple diffusion model based on Fick’s time-dependent law. This model can provide the designer with a time-dependent spatial distribution of the potential diffusion and therefore of the embrittled material. This idea is, per se, not original. An equivalent diffusion coefficient model has already been developed and is available in the open literature [12]. Such an equivalent diffusion model, implemented in Abaqus 6.14 [13], can calculate the distribution of diffused oxygen. The model has been validated and calibrated with existing weight gain data published by Detwiler and Opila [14]. This model is relevant for the type of CMCs targeted in this work: SiC/BN/SiC. In other words, this material system is made with SiC fibres, a SiC matrix, and an interface of boron nitrate (BN). The toughening mechanism of this type of material is given by the BN interface surrounding the fibres. Cracks will preferably propagate along this interface [15]. There are two more reasons for adding a BN interface. The first one is to help relax the compressive residual stresses [16], and the second is to have a self-healing mechanism against cracks from oxidation and the forming of a boron–silicate glassy phase [17].

Gaseous species in the engine environment, including oxygen from excess air and water vapor from combustion, result in an environment that is detrimental to a CMC. Detwiler and Opila’s study considers reactions (and therefore weight uptake experiments) between both 100% O_2_ (dry oxygen) and 50% H_2_O/50% O_2_ (wet oxygen) and a SiC/BN/SiC CMC. Chemical reactions start after the oxygen (and/or water) are diffusing inside the material. Data concerning water are not considered in this paper, and they are part of future work. Chemical reactions of oxygen are described as follows. At 800 °C in dry oxygen, a silica (*SiO*_2_)-rich glass forms on the surface of the CMC per (1):

(1) $$\frac{2}{3}SiC+O_{2}\left(g\right)=\frac{2}{3}SiO_{2}+\frac{2}{3}CO(g)$$

Boron, from the *BN* fibre coatings, oxidises to form a condensed liquid phase via (2) and (3), respectively:

(2) $$\frac{4}{3}BN+O_{2}\left(g\right)=\frac{2}{3}B_{2}O_{3}(l)+\frac{2}{3}N_{2}$$

(3) $$\frac{4}{3}B+O_{2}(g)=\frac{2}{3}B_{2}O_{3}(l)$$

Liquid boria can volatilise at elevated temperatures, per (4).

(4) $$B_{2}O_{3}(l)=B_{2}O_{3}(g)$$

The resultant silica and boria can mix to form a borosilicate glass, given by Equation (5):

(5) $$xB_{2}O_{3}+ySiO_{2}=xB_{2}O_{3}\times ySiySiO_{2}(l,g)$$

The equivalent diffusion model proposed in this paper, being a holistic approach for design purposes, does not consider the single CMC constituent oxidation but a uniform distribution (valid if the material system has a regular architecture). It needs to be completed with a reaction rate equation. Weight uptake data, in fact, are only a first part of the full picture. Once oxygen diffuses in the material, reactions (1–5) start. Reaction rates are defined as per Equation (6), and they are applied both to SiC and BN.

(6) $$\frac{d}{dt}\left(\frac{\rho_{c}}{\rho_{c}^{0}}\right)=k_{0}exp\left(\frac{{-E}_{a}}{RT}\right)p_{ox}^{n}$$

where

*ρ^0^_c_* is the initial concentration of the element;
*ρ_c_* is the actual concentration of the element;
*p_ox_* partial pressure of oxygen;
*k*_0_, *R*, *n* constants;
*T* temperature.

Reactions and therefore embrittlement are therefore a function of oxygen partial pressure (and therefore its concentration in the air) and temperature. In this work, as temperature and partial pressure are constants of Detwiler and Opila’s test, their influence cannot be investigated or captured yet.

Weight uptake experimental data will be reproduced with a simple time-dependent Fick’s equation, and the calibration process is described in Section 2 of this article. Diffusion data, and the consequent chemical reactions, are contributing to the material embrittlement. This is of relevance when evaluating the structural integrity of the material system subjected to static (but still applied for a period of time) and time-dependent loads. Stiffness material data of a stress model need therefore to be calibrated to make the stiffness not only a function of the temperature but also of the concentration of reaction products. However, this is not the only coupling that exists. In fact, crack opening caused by the stress field accelerates the diffusion process observed in an unloaded specimen. In this work, the diffusion coefficient has been modelled as damage dependent. The damage is calculated as a function of the strain field produced by the mechanical load applied.

The concept of the time-dependent degradation of CMCs materials is well documented in the open literature. Mital et al. have modelled the stiffness degradation as a function of time [18]. The authors remark that several explanations are suggested about the oxidation-driven stiffness degradation. One hypothesis is that boron nitride (BN) coating deposited on the fibres oxidises, causing a fusion of fibres. Another hypothesis assumes that the SiC fibres are oxidised, forming a silica scale leading to premature fibre failure. A theory also exists, which is independent from any oxidation hypothesis, stating that the SiC fibre strength is intrinsically time dependent due to the slow crack growth of flaws in the fibres. The time-dependent stiffness degradation is something not strictly related to fatigue [19]. Even without load cycle accumulation, damage is observed in a form of stiffness degradation even for static loads with a dwell time. This is analogous to creep damage in metals. Work performed by the US Air Force Research Laboratory [20] and NASA [21] are providing supporting information in this direction. It is worth mentioning that the inherent defects and porosity of the material accelerate the time-dependent damage [22].

It must be remarked that the problem of stiffness and strength degradation caused by oxidation has been studied for decades, even in metallic material systems [23]. The oxidation and relative structural degradation of a complicated material system, such as the ceramic matrix composite, has been studied at the constituents’ level in detail in [24]. Alkaline corrosion of SiC fibres has also been investigated in [25]. It is important to consider that minor elements are very often present in the air, in particular sodium for on-board planes and airports near the sea. For what concerns SiC fibre-reinforced SiC matrix composites with a BN interphase (SiC/BN/SiC), NaCl ingested into the engine in marine environments can react with sulphur impurities present in fuel, forming molten sodium sulphate deposits, resulting in hot corrosion [26,27]. The reader can also find the relevant chemical reactions in [26,27]. The hot corrosion of bulk SiC in a sodium-rich environment has, however, not been investigated in this paper or in the experimental work [14] used as the basis of this document.

The structural assessment computational framework proposed in this paper is performed with two distinct models, one structural (stress model) with mechanical loads applied and one diffusion model, able to calculate the oxygen diffusion through the material. At every iteration, the structural stiffness is degraded as a function of the previous time-step concentration and as a function of time. With such a degraded stiffness, the damage is calculated as a function of the total strain. Lastly, the diffusion model is updated in such a way that the diffusion coefficient is a function of the damage. This iterative procedure is repeated at any user-defined time step.

The time step of the iteration has been varied until convergence. The computational procedure is described in detail as well as the results of some test cases in Section 3. The final goal of this procedure is to monitor both the oxygen diffusion as a function of time as well as the stiffness degradation and consequent damage progression.

In the authors’ opinion, this computational approach could be extended to the moisture-induced damage in the organic matrix composites [28].

## 2. Materials and Methods

**Figure 1:**
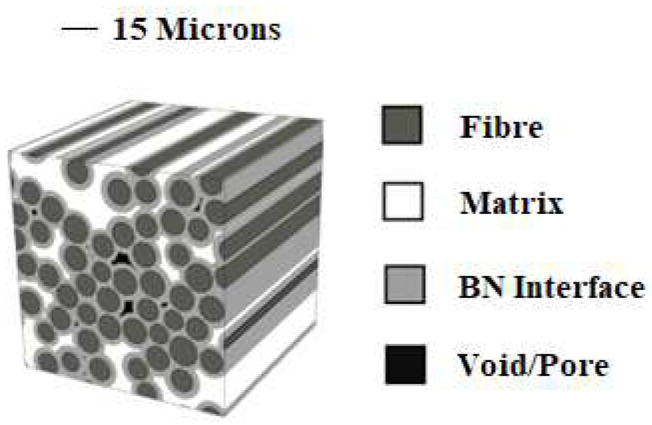
*BN* interface surrounding the fibres [15].

The material experimental data of oxygen uptake for unstressed specimens have been produced by Detwiler and Opila [14]. Their work specifically concerns SiC/BN/SiC. This material system has been chosen because the toughening mechanism is designed to facilitate the crack propagation along the BN interface surrounding the fibres. An image of the architecture giving an illustration of the toughening method is shown in Figure 1 [15]. It is assumed that the weight uptake is directly proportional to the reaction products, causing embrittlement. The paper presents results of dry oxygen and wet oxygen weight uptake. In this work, only the dry oxygen weight uptake was considered.

Main goal of this part of the work was to find an equivalent diffusion coefficient able to minimise the error between the experimental data produced and simulation results (Fick’s equation only [29]) for 800 °C and 600 °C.

### 2.1. Diffusivity Analysis via Thermal Analogy

**Figure 2:**
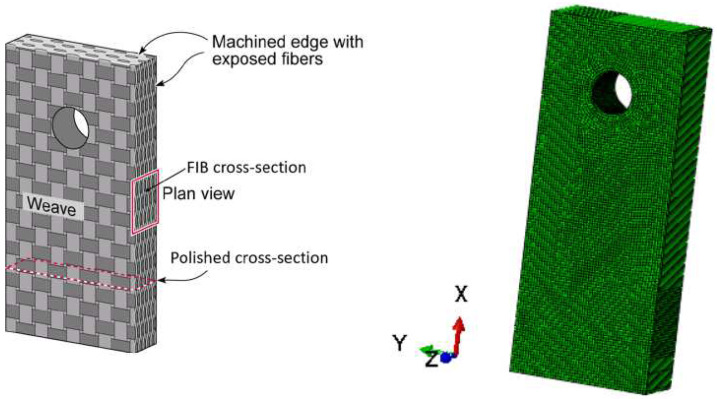
Weight uptake specimen taken from the work of Detwiler and Opila [14].

The specimen exposed in open air at 600 and 800 degrees Celsius is shown in Figure 2. Coupon dimensions are 25.4 mm × 10.2 mm × 4.5 mm. A 3.175 mm diameter hole was machined mm from the top edge of the sample to suspend the sample whilst exposing it. The material is a SiC/BN/SiC composite with a multilayer 5HS architecture. To perform both diffusion and structural simulations, an FE mesh has been created with 136,752 HEX20 elements.

Nine engineering constants have been used to characterise the elastic behaviour of the composite in line with the work performed by Canale et al. [29]. In this work, as two relatively cold temperatures were analysed, at least in terms of high temperature applications, such as an aero-engine turbine sub-system, a unique set of stiffness values are given and reported in Table 1.

Mass diffusion simulations have been performed using a thermal conductivity problem analogy. Just to give the reader an example of this analogy, let a simple 1D diffusion equation be written as follows (7):

(7) $$\frac{\partial C}{\partial t}=D\frac{\partial^{2}C}{\partial x^{2}}$$

where

*C* is the moisture concentration;
*t* is the time;
*x* is the spatial coordinate (just one coordinate in this example);
*D* is the diffusion coefficient (isotropic, in this example).

Let this equation be compared with a simple 1D thermal conductivity in Equation (8):

(8) $$\frac{\partial T}{\partial t}=\frac{k}{\rho c}\frac{\partial^{2}T}{\partial x^{2}}$$

where

*ρ* is the material density;
*k* is the thermal conductivity;
*c* is the specific heat.

This implies that a thermal conductivity analysis can be performed to simulate mass diffusion as long as the following condition (Equation (9)) is respected:

(9) $$\frac{k}{\rho c}=D$$

**Figure 3:**
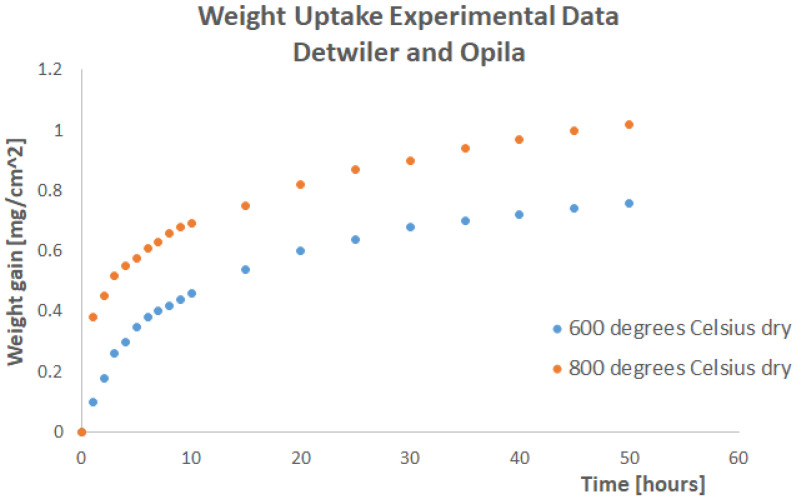
The Weight Gain Experimental Results for the Geometry of Figure 2.

The factor *ρc* was set to unity in the simulations performed for this paper. If the material density was *ρ* = 2.8 × 10^−9^ Tonnes/mm^3^, the specific heat was set equal to 3.57143 × 10^8^ mJ/Tonnes/K. Mass uptake simulations were performed in Abaqus to predict the dry oxygen weight uptake experimental curves at 800 °C and 600 °C. The experimental results are shown in Figure 3.

**Figure 4:**
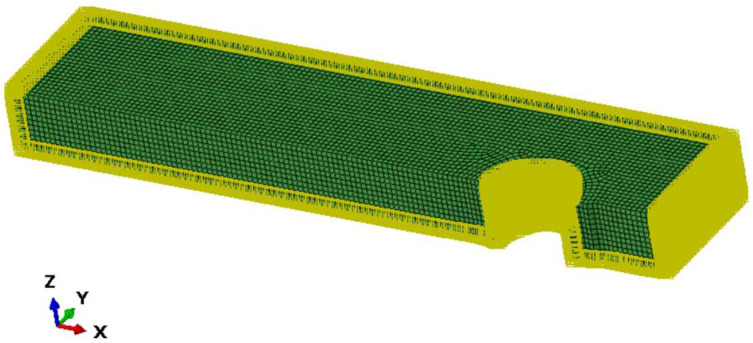
A quarter of the model is shown. Saturation boundary conditions are applied to the external surface. This is an imposed temperature of 1.03.

Thermal conduction Abaqus model wasn set with a saturation condition as a boundary condition on the external surfaces, as shown in Figure 4. This saturation is temperature, as temperature is basically the measure of concentration in this kind of simulation.

The only parameter to tune to calibrate this diffusion approach to model oxidation was the thermal conductivity *c*. This parameter was optimised in such a way to minimise the error between the experimental uptake curve *u*(*t*) and the simulation update curve *s*(*t*). In mathematical terms, the error *e* is defined as per (10):

(10) $$e=\frac{\int_{0}^{t}\left|u\left(t\right)-s(t)\right|dt}{\int_{0}^{t}u\left(t\right)dt}$$

A DoE (design of experiment) of automatic simulation and data post-processing [6] was coded in Matlab to find a value of conductivity *c* able to provide an error below 2%.

### 2.2. Oxydation Diffusion Model Calibration

**Figure 5:**
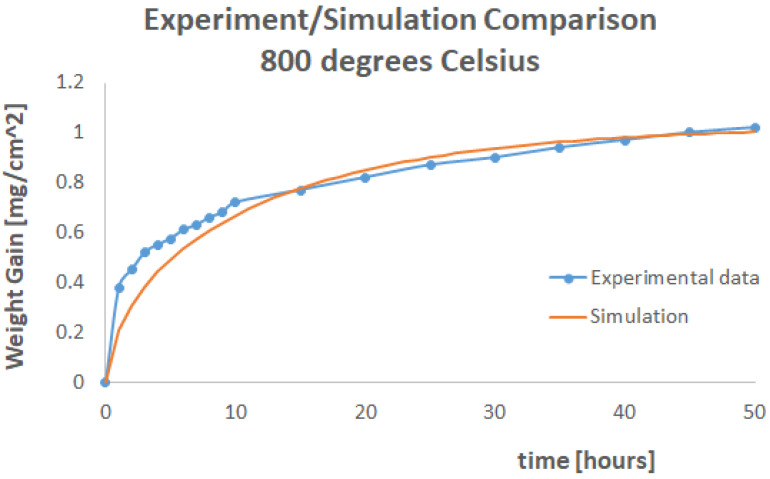
Experimental and Simulation Curves for 800 °C.

Weight gain results for 800 °C are given in Figure 5.

**Figure 6:**
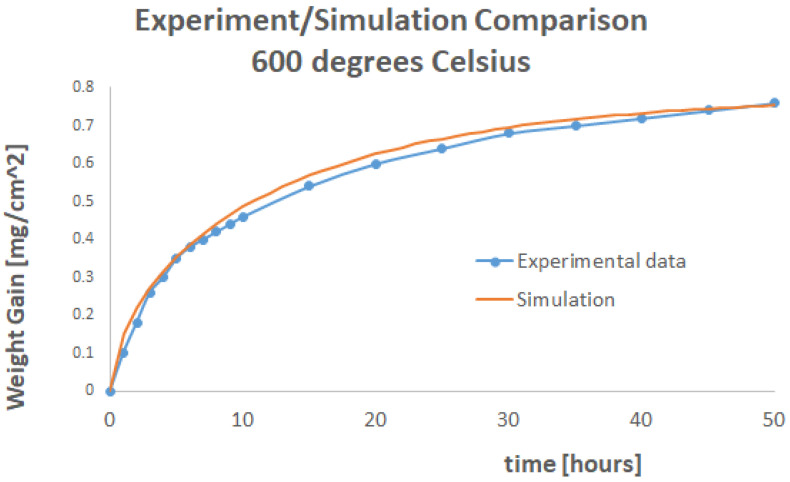
Experimental and Simulation Curves for 600 °C.

Weight gain results for 600 °C are given in Figure 6.

For both these simulations, a conductivity of 0.1 mW/mm/K was found as an optimum to minimise the error between the simulation and experimental curves.

At 600 °C, the shape of the curve produced via simulation and the experimental one were extremely close. The physics were reproduced with a high level of fidelity. At 800 °C, although the error was minimised, the model tended to overpredict weight uptake for the first 15 h of the experiment before falling in line with the experimental data. This may be an indication of chemical reaction volatilisation.

### 2.3. Iterative Procedure to Couple Stress and Diffusion Models

Methods and calibration presented in Section 2.2 of this paper were valid for an unstressed specimen exposed to open air with oxidation happening (due to oxygen only). When a stress field is applied, the induced microcracking, or even visible damage, accelerates the oxidation process. On the other hand, when oxygen diffuses through the CMC, causing the chemical reactions described in Section 1, embrittlement occurs beside the measured weight gain. The CMC, as the oxidation progresses, becomes more compliant (the stiffness degrades with time) and prone to failure. This coupled behaviour was implemented into an iterative and fully automatic computational framework, described in this section. Results of numerical simulations are presented in Section 3.

#### Assumptions

**Figure 7:**
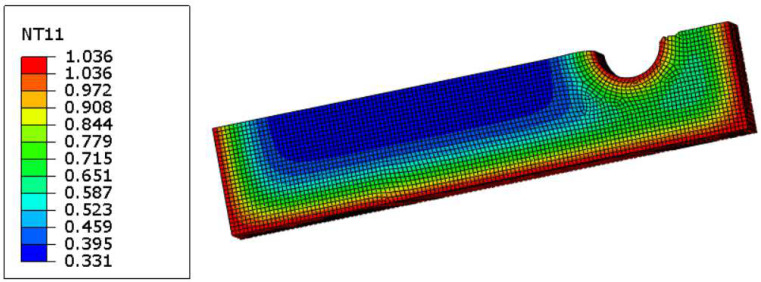
An example of the spatial distribution of the material oxidation (modelled as a temperature) at a given time of the oxidation process.

There were two models needed for the iterative procedure: one diffusion model, modelled via thermal conductivity, as previously discussed, and a structural model. The diffusion model was run in such a way to generate an oxidation spatial distribution as a function of time, as per example shown in Figure 7.

A spatial distribution of the oxygen concentration as a function of time, and therefore of the embrittlement, was used iteratively in the structural model. It was assumed that the reaction products at the given temperature of the test were directly proportional to the oxygen concentration. If temperature variation across the mechanical component had to be considered as per (6), the reaction rates shall be calculated as a function of temperature and partial pressure of oxygen in the atmosphere. For the sake of providing the reader with the iterative computational framework, thermal gradient and dependency from the oxygen partial pressure have not yet been introduced in the computational framework and will be part of the future development of the code. The updated code will be validated when experimental data are available.

A linear time-dependent stiffness degradation was implemented in this work. This was only an assumption in line with the creep data of the open literature [30,31]. However, different CMC systems may need to adapt different assumptions. This does not affect the validity of the computational framework. The stiffness degradation was not only time dependent but it was also accelerated by the oxidation content. In other words, stiffness had a linear dependency on the oxygen concentration as well as time. This was an assumption backed up by the work of Santosh et al. [22].

At a given temperature, in our case, 800 °C, it is assumed that the embrittlement (due to chemical reactions) is simply proportional to the oxygen concentration [11]. This was modelled as a linear decay in stiffness proportional to the concentration of oxygen, which was calculated with the diffusion simulation. In other words, the stiffness as a function of oxygen concentration is given in Table 2 and Table 3. The stiffness was kept unchanged up to 20% of the saturation value of oxygen, then it was linearly degraded as a function of the oxygen concentration. This 20% threshold was arbitrary, and it is a parameter that the user can calibrate as long as the experimental data are available. Between the beginning of the exposure and 20 h, consistent with the data published by Santosh [22], if the oxygen exceeded 20% of saturation, a linear stiffness degradation with time was applied.

The CMC is also characterised by pseudo-plastic stress–strain behaviour. The non-linearity is well described by Alabdullah et al. [30]. Analogous stiffness degradation as a function of time and oxygen concentration were applied. The data used for the proposed computational framework are reported in Table 4 and Table 5.

**Figure 8:**
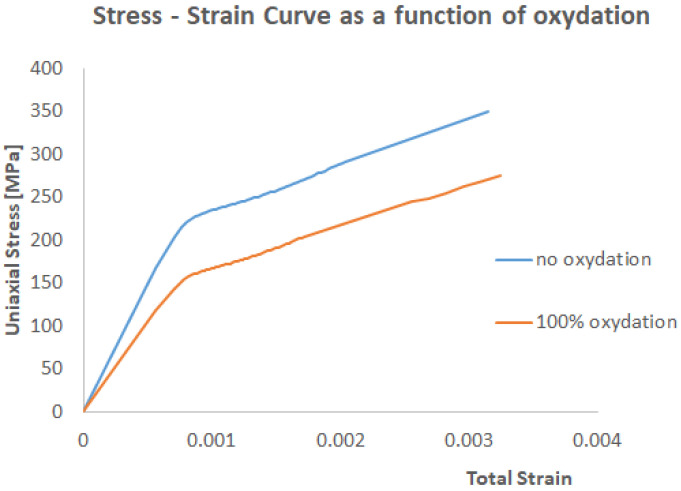
An example of stiffness degradation (800 °C, t = 0) as a function of time and oxygen concentration.

An illustration of the degradation of a function of the oxidation and time is shown in Figure 8.

**Figure 9:**
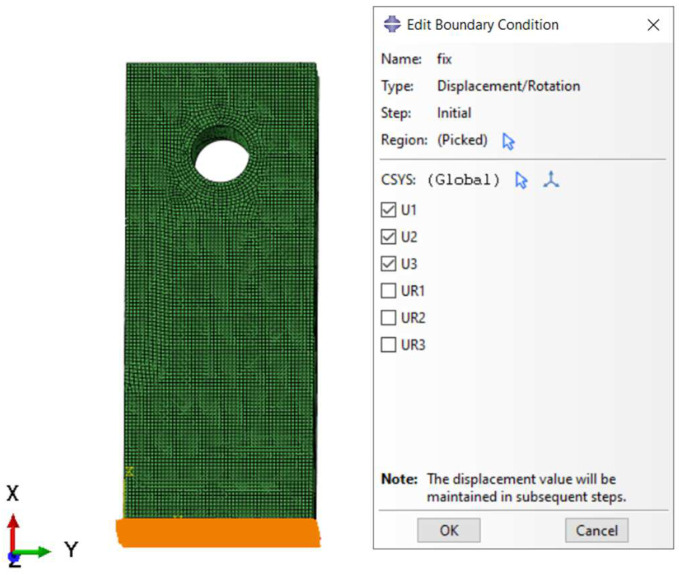
The boundary condition applied to the stress model. The arrows are a graphical representation of the Boundary Condition in Abaqus 6.14.

**Figure 10:**
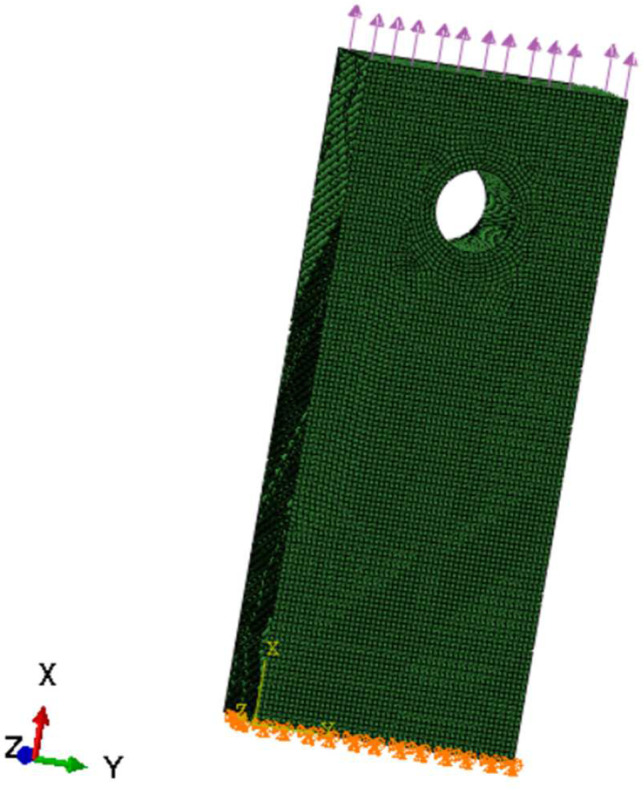
The applied load as a negative pressure. The orange symbol is a representation of the Boundary Condition in Abaqus 6.14. The arrow is a graphic representation of the load.

Once the material properties were updated as a function of oxidation, the stress model was used to re-calculate the strains. The stress model used to validate the proposed computational framework is shown in Figure 9 and Figure 10, where fixed boundary condition and applied load are shown.

Once the stress model gave its results in terms of stress and strain distribution, damage was calculated. A CDM (continuous damage mechanics) [31] theory could be used and integrated in the computational framework. This, however, would slow down the iterations. Since the aim of this work was mainly to demonstrate the validity of the computational framework, a conservative strain criterion was therefore used. The Von Mises total strain giving the unitary damage was 0.0015 [32]. In other words, it was as follows:

(11) $$\varepsilon_{0}=0.0015$$

The damage, *D*, is not in structural terms but rather as a “crack density” parameter to be correlated with the oxidation diffusion. In other words, the damage is calculated at each node of the structural part of the simulation with (12):

(12) $$D=\frac{\epsilon }{\epsilon_{0}}$$

where

*ε* is the Von Mises strain at each node.

Once the damage is calculated, the iteration procedure can come back to the diffusion model. Thermal conductivity, the parameter driving the oxygen diffusion in the simulation, was adjusted at every iteration as a function of damage, as per Table 6.

The diffusion analyses could then be re-started from the previous time step (Abaqus re-start). In the programming solution chosen in this work, a new analysis step was simply defined, progressing the analysis from the simulation time where it was left. The equivalent diffusion oxidation model simulation time progressed until results were exported, and a cycle was complete. The procedure was iteratively repeated until the full time of the simulation was reached. Convergency studies were performed on the largest operational iteration time step (time at which the diffusion and the stress model exchange information) possible, suggested for this type of iterative procedure.

## 3. Results and Discussion

**Figure 11:**
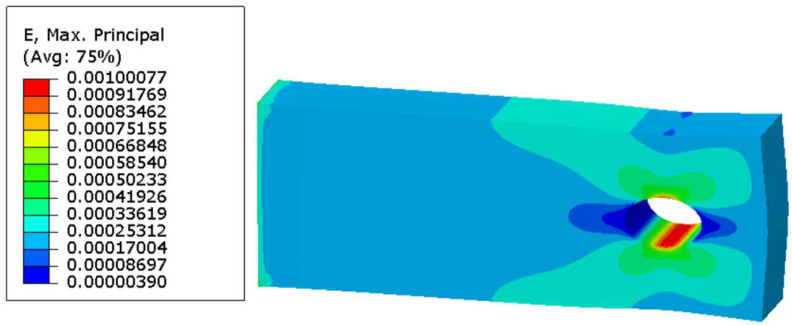
The illustration of the location of the maximum total strain of the stress model employed to verify the computational framework.

A 50 h simulation has been performed at 800 degrees Celsius with an applied stress (nominal) of 50 MPa. There are two relevant performance measures to be monitored and recorded as a function of the simulation time: oxygen diffusion as a function of time and total strain (peak) as a function of time. An illustration of the total strain plot of the specimen subjected to a constant nominal pressure is shown in Figure 11.

**Figure 12:**
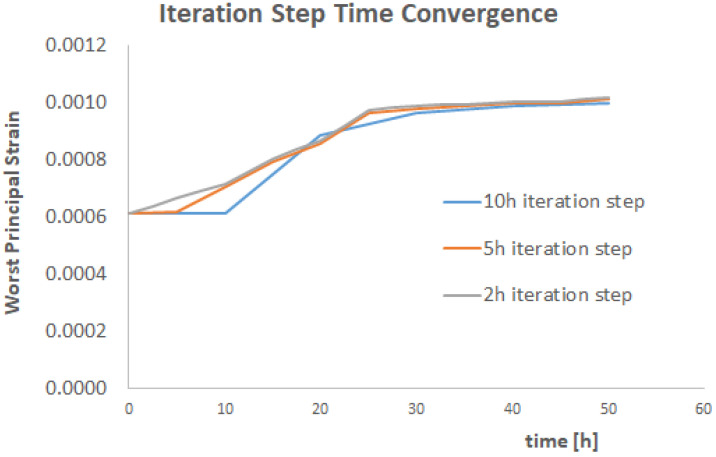
The total strain evolution during the iterative procedure for three different iteration times.

**Figure 13:**
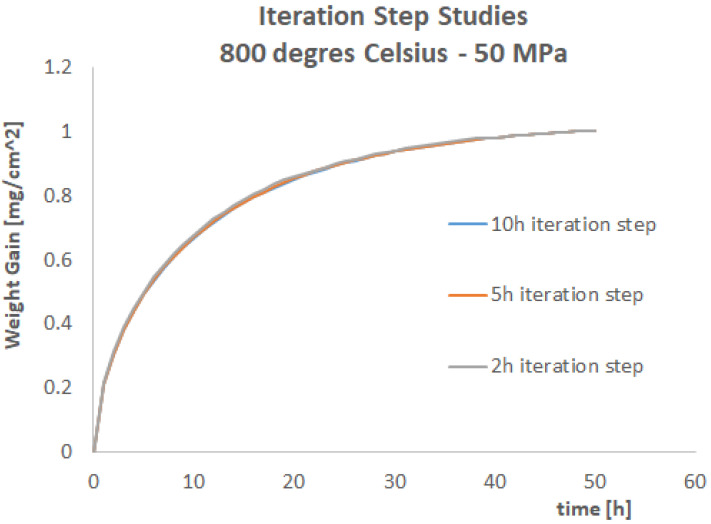
The weight uptake evolution during the iterative procedure for three different iteration times.

Three different iteration time steps have been investigated: 10 h, 5 h, and 1 h time steps. The results of these three simulations are shown in Figure 12 and Figure 13. A fourth simulation with a 1 h time step has been performed, and the results are identical to the 2 h step. This means that the 2 h step is the maximum iteration time step to obtain a converged and stable solution.

It can be observed that the results are collapsing towards the same values for the 5 h and 2 h steps (as remarked, the 1h step simulation gives identical results to the 2 h step simulation), with the only exception the first 5 h of the strain evolution. Consequently, for engineering purposes, the 5 h iteration step can be considered good enough to make predictions. Another argument in favour of this suggestion is the computational time. A 50 h simulation with a 5 h iteration step has been running for ca 75 h using 40 CPUS clusters at the University of Derby (Derby, UK). This time increases to ca 180 h when reducing the iteration step to 2 h. Given that the shape of the diffusion curve is basically not changing and given that a precision of the 2 h step is improving only the strain history in the very first 5 h of the simulation, the best operative option could be to have small step iterations of 1 h/2 h at the beginning of the simulation (for the first 15 h) and then increase the simulation time to 5 h or even 10 h toward the end of the simulation. This kind of scheme has not been tried, and it is left to the reader as a part of future work on this topic.

**Figure 14:**
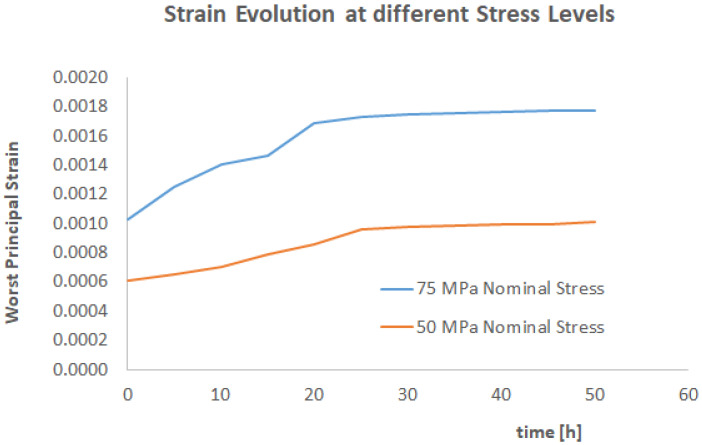
The total strain evolution as a function of time for 75 MPa applied pressure.

**Figure 15:**
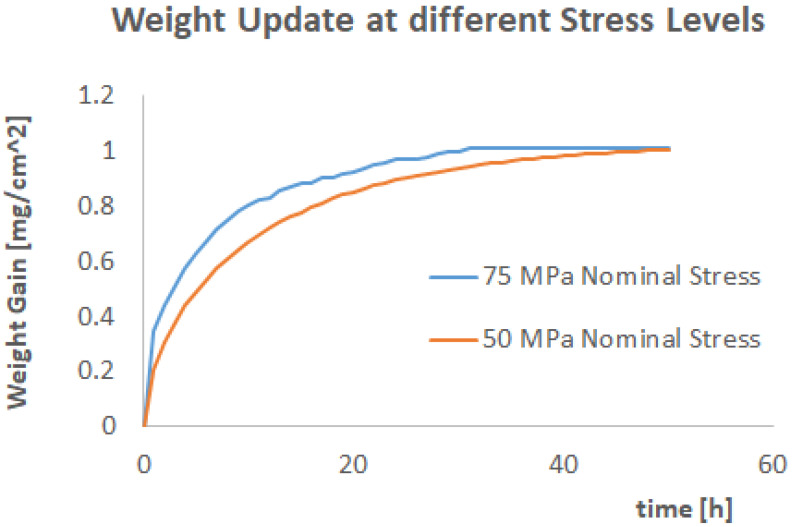
The weight uptake evolution as a function of time for 75 MPa applied pressure.

In the authors’ view, one more question needs to be answered to prove the effectiveness of the proposed computational framework, which is about the effects of increased stress. For this reason, the simulation has been repeated for a 75 MPa nominal stress (and nominal pressure applied). The results are shown in Figure 14 and Figure 15.

As expected, an increased stress accelerated the weight uptake. The strain history (and therefore the damage evolution) is accumulating much faster than the 50 MPa case.

## 4. Conclusions

Embrittlement due to oxidation, besides the challenges in manufacturing, is one of the main challenges for an industrial mass deployment of CMCs in the civil aero-engine industry. Two separate aspects of this peculiar CMC problem have been tackled in this paper. The first one was to reproduce oxygen weight uptake experimental data produced with a simple Fick’s law implemented in the commercial tool: Abaqus. The parameters of the calibration are given to the reader to minimise the error between the simulation and the experimental data. The shape of the weight uptake curve has been reproduced with a high level of fidelity at 600 °C. A slight difference in curve shapes exists at 800 °C, but the error is still below 2%. The second part of the work proposed in this paper is a design iterative computational framework (implemented in Matlab and Abaqus) for iteratively calculating the mass uptake and strain distribution of CMC structures subjected to a time-dependent mechanical loading. The stress field arising on a CMC component, in fact, increases micro-cracking and therefore facilitates oxygen diffusion. On the other hand, when oxygen diffuses, chemical reactions produce embrittlement and consequent stiffness degradation. Oxygen diffusion and stress are therefore coupled. The problem has been tackled with an iterative procedure, where the stiffness is degraded both as a function of time and oxygen concentration. Sensitivity studies have been performed on the iteration step time as to understand the balance between the precision of the results and the computational time. Sensitivity studies on the applied loads have also been performed. It is worth remarking that the proposed iterative procedure may be adapted to organic matrix composites undergoing moisture absorption. The mechanisms of stiffness degradation are analogous for what concerns the prospective computational framework (an adaptation of the degradation assumption must be obviously adapted).

As a part of future work, the effect of temperature gradients and partial oxygen pressures must be understood and implemented in the computational framework. This work will be also extended to the water/oxygen ingestion of the different chemical reactions driven by water/oxygen/CMC mixtures.

It must also be remarked here that the experiment, being a first step of a long research program, is quite simplified (constant temperature, water, and oxygen ingress only). In a real flight profile, the temperature gradients and potential non-uniform cracking in the coating system play a big role in oxidation and consequent stiffness degradation. Furthermore, oxygen and water are not the only molecules responsible for oxidation. Sodium also plays a big role, and the authors are fully aware of this limitation. All these aspects will be considered as a part of future work.

## Figures and Tables

**Table 1:** The elastic properties of the SiC/BN/SiC composite used for this work. The stiffness degradation between 800 °C and 600 °C has been considered small enough to be neglected.

*E_11_* [MPa]	*E*_22_ [MPa]	*E*_22_ [MPa]
300,000	300,000	260,000
***G*_12_ [MPa]**	***G*_13_ [MPa]**	***G*_23_ [MPa]**
140,000	120,000	120,000
***v*_12_**	***v*_13_**	***v*_23_**
0.15	0.15	0.15
***ρ* [Tons/mm^3^]**		
2.8 × 10^−9^		

**Table 2:** Elastic data as a function of oxygen concentration and therefore as a function of oxidation at the initial time (t = 0) and 800 degrees Celsius.

Elastic Property (t = 0 h)	0–20% Oxygen	100% Oxygen
*E*_11_	300,000	240,000
*E*_22_	300,000	240,000
*E*_33_	260,000	208,000
*G*_12_	140,000	112,000
*G*_13_	120,000	96,000
*G*_23_	120,000	96,000

**Table 3:** Elastic data as a function of oxygen concentration and therefore as a function of oxidation after 20 h of exposure.

Elastic Property (t = 20 h)	0–20% Oxygen	100% Oxygen
*E*_11_	300,000	204,000
*E*_22_	300,000	204,000
*E*_33_	260,000	176,800
*G*_12_	140,000	95,200
*G*_13_	120,000	81,600
*G*_23_	120,000	81,600

**Table 4:** Plastic data as a function of oxygen concentration and therefore as a function of oxidation at the initial time.

Oxygen Content Dependency t = 0 h	Stress [MPa] 0–20% Oxygen	Plastic Strain 0–20% Oxygen	Stress [MPa] 100% Ox	Plastic Strain 100% Oxygen
	167	0	134	0
	350	0.0013839	280	0.0013839

**Table 5:** Plastic data as a function of oxygen concentration and therefore as a function of oxidation after 20 h.

Oxygen Content Dependency t = 20 h	Stress [MPa] 0–20% Oxygen	Plastic Strain 0–20% Oxygen	Stress [MPa] 100% Ox	Plastic Strain 100% Oxygen
	167	0	114	0
	350	0.0013839	240	0.0013839

**Table 6:** Conduction as a function of damage.

Conductivity [mW/mm/K]	Damage
0.10	0
0.10	0.2
0.17	1

## Data Availability

The original contributions presented in the study are included in the article, further inquiries can be directed to the corresponding author.
